# Sleep quality and injury risk in adolescent football players: an amplified effect for high-risk athletes revealed by quantile regression

**DOI:** 10.3389/fpubh.2026.1799229

**Published:** 2026-05-28

**Authors:** Nanhu Li, Zhengri Quan, Dan Pang, Donghui Jin

**Affiliations:** 1 School of Physical Education, Kookmin University, Seoul, Republic of Korea; 2 School of Physical Education and Health, Changchun Normal University, Changchun, China; 3 School of Sports Science, China Three George University, Yichang, China

**Keywords:** adolescents, athletic injuries, effect heterogeneity, football, precision prevention, quantile regression, sleep

## Abstract

**Background:**

Sleep quality is a key regulatory factor in sports injury, but it is not clear whether its association with injury risk is homogeneous or varies among athletes with different baseline risks. This study explored the heterogeneous and non-linear associations between sleep quality and risk of impairment.

**Methods:**

We conducted a cross-sectional analysis of 478 young football players from 12 Chinese clubs. Sample size was determined based on the need to provide stable estimates across multiple conditional quantiles, exceeding the general rule of thumb for complex multivariate models. Sleep quality was assessed using the Pittsburgh Sleep Quality Index while recording its subcomponents, such as sleep duration. Injuries, including time lost and severity, were recorded during the past season. Using quantile regression, we analyzed the association between total PSQI score and the number of impairments across the conditional distribution (*τ* = 0.25, 0.50, 0.75, 0.90) after controlling for team-level fixed effects and covariates.

**Results:**

We observed a strong positive correlation between the PSQI score and the incidence of injury. Quantile regression revealed significant heterogeneity: this adverse association was greatly amplified at higher quantiles of injury risk. The PSQI coefficient increased from 0.07 (95% confidence interval: 0.01, 0.13) in the 25th quantile to 0.33 (95% confidence interval: 0.23, 0.43) in the 90th quantile, indicating that the strength of the association was enhanced about fivefold for high-risk athletes. Separate analysis for severe injuries corroborated this finding. A nonlinear J-type dose–response relationship was also identified, with the risk accelerating when the PSQI score exceeded 6.

**Conclusion:**

The association between poor sleep and injury risk was not uniform but showed a clear gradient, with the most significant adverse associations concentrated in high-risk athletes. This quantile-dependent effect highlights the potential of sleep intervention as a precision strategy. While sleep quality remains important for all adolescent football players, targeted sleep health promotion for high-risk individuals can maximize the injury prevention benefits of adolescent athletes, but prospective studies are still needed to establish causality.

## 1 Introduction

Football is one of the most popular sports among youth worldwide, providing a wide range of physical, social, and psychological benefits (1). However, participation in football also comes with a considerable risk of sports injuries (2). These injuries can trigger a range of adverse outcomes, from immediate pain and dysfunction, to long-term musculoskeletal sequelae, psychological distress, and increased financial burden on the healthcare system (3). Therefore, developing effective and evidence-based injury prevention strategies has become a key public health priority in youth sports (4).

Among the many identified risk factors, sleep has emerged as a crucial and regulatable determinant of exercise performance and health (5). As a fundamental physiological process, sleep is indispensable for cognitive recovery, memory consolidation, metabolic homeostasis, and neuromuscular recovery (6). Extensive epidemiological evidence has now established that poor sleep quality and short sleep duration are independent risk factors for sports injuries (7). While previous studies have explored the multifaceted benefits of adequate sleep on physiological and cognitive performance measures critical to recovery and damage resistance, the specific contributions of different sleep dimensions, such as duration, warrant further investigation. The underlying mechanistic pathways are manifold, including impaired reaction time and cognitive function, reduced motor learning and skill acquisition, dysregulated hormone levels (e.g., elevated cortisol and decreased growth hormone), and increased systemic inflammation (8). Traditionally, epidemiology to study this relationship has relied heavily on regression models based on means, such as ordinary least squares regression or logistic regression (9). These methods, while extremely valuable in identifying population-averaged effects, are essentially limited to estimating a single “average” effect of sleep on injury risk, and implicitly assume that this effect is uniform across all athletes (10).

Considering the complex and multifactorial etiology of sports injuries, this assumption of homogeneity is likely to be untenable. The “complex systems” approach to sports injuries considers that injuries are not caused by a single factor, but by the nonlinear interaction of multiple risk factors and vulnerabilities (11). Applying this theoretical perspective to sleep, we hypothesize that poor sleep is not isolated, but rather synergistically amplifies an athlete’s existing risk profile. This concept, which we call “vulnerability amplification,” suggests that athletes who are already vulnerable to injury due to high training loads, previous injury history, or subclinical physiological dysregulation will be disproportionately more negatively affected by poor sleep than their low-risk peers. A key and unresolved question persists: Is the association between poor sleep and injury risk uniform across the athlete population, or is it heterogeneous in effect, meaning that the association may be amplified for athletes who are already at higher risk? The question goes beyond “is sleep important” to “who is most important.” Traditional analytical methods have difficulty in detecting such heterogeneity, may mask the true nature of the relationship, and lead to ineffective “one-size-fits-all” prevention recommendations (12). This gap highlights the urgent need for an analytical framework capable of simulating the full conditional distribution of outcomes (13).

Quantile regression is a powerful and innovative methodological alternative that directly addresses this limitation (14). Unlike traditional regression that models the conditional mean, quantile regression models the conditional quantiles (e.g., median, 90th percentile) of the outcome distribution. This allows a comprehensive examination of how the relationship between exposure factors and outcomes varies at different points in the distribution of outcomes (15). In the context of injury risk, it enables direct exploration of whether the association of sleep quality with injury is stronger for athletes born at high risk (high quantile) than for athletes born at low risk (low quantile) (16).

The primary objectives of this study were threefold: (1) to examine the global association between sleep quality, as measured by the Pittsburgh Sleep Quality Index, and risk of injury in a multi-district sample of adolescent football players; (2) analysis of the potential heterogeneity of this association across the conditional distribution of injury risk utilizing quantile regression, with a particular focus on identifying disproportionately strong associations in high-risk adolescent athletes (high quantiles); and (3) exploration of the potential non-linear dose–response relationship between PSQI scores and injury risk. We hypothesized that the deleterious association between poor sleep quality and injury risk was not uniform but was significantly amplified in athletes with high baseline injury risk. By quantifying this potential effect heterogeneity, this study aims to shift the paradigm from generalist recommendations to precision prevention strategies, identifying which athletes would benefit most from sleep-focused interventions.

## 2 Methods

### 2.1 Study design and participants

This study was a multicenter cross-sectional analysis, with data collected during the 2024 competitive season (March to November) using a retrospective cohort framework. Convenience sampling method was used to recruit athletes from 12 professional youth football clubs representing Northeast China, North China, East China, South China and Southwest China. This national coverage ensures that the sample is broadly representative of different socioeconomic and training environments. Following the general recommendation for multivariate regression models to have at least 10–15 observations per predictor, and considering the need for stable estimates in extreme quantiles (e.g., *τ* = 0.90), a sample size of more than 400 was considered adequate for our preliminary analysis. Our final sample of 478 athletes met this requirement. Inclusion criteria included: (1) age 12–18 years; (2) valid registration with a participating club for at least 6 months; (3) participation in systematic football practice at least three times a week; and (4) written informed consent from the player and his/her parent/legal guardian. Exclusion criteria included: (1) confirmed sleep disorder; (2) major musculoskeletal injury or surgery within 3 months prior to data collection; (3) neurological or psychiatric illness that affects sleep or perception of risk of injury; and (4) use of medications that significantly affect sleep patterns.

### 2.2 Variables and measurement

#### 2.2.1 Primary outcome: injury incidence

Injury data were collected through an integrated surveillance system that combines standardized self-administered questionnaires and validation of club medical records. We use an operational definition consistent with the international consensus on the epidemiology of football injuries (17): it refers to any physical discomfort that occurs during football training or competition, resulting in (a) the inability to complete the current training/competition, or (B) the absence of at least one subsequent planned training or competition. For each injury event, record details including date of occurrence, mechanism (traumatic or straining), body part, specific diagnosis, and length of absence. The primary outcome variable was operationalized as the total number of injuries resulting in time loss sustained by each athlete over the entire observation period (past one full season). The second outcome is a serious injury, divided into two categories, that is, any injury that results in the inability to fully participate in training and competition for more than 28 days. To improve data accuracy, we used the triangulation method to cross-validate self-reported injuries with club medical records and coach logs (18). The clubs studied employed team doctors who maintained standardized medical records. When there was a discrepancy between the self-report and the medical record, the more conservative estimate from the medical record was used by default after a detailed re-interview of the athlete and coach for adjudication.

#### 2.2.2 Core exposure: sleep quality assessment

Sleep quality was assessed using the validated Chinese version of the Pittsburgh Sleep Quality Index (19). The PSQI is a 19-item self-rating instrument that assesses sleep quality and disorders over the past month. The PSQI produces seven component scores: subjective sleep quality, time to sleep, sleep duration, habitual sleep efficiency, disturbed sleep, use of sleep medication, and daytime dysfunction. These component scores were added to yield a global PSQI total score of 0–21, with higher scores indicating poorer sleep quality. An established clinical cutoff value of >5 was used to identify participants with significant sleep disturbances (20). The Chinese version of PSQI showed good psychometric properties in adolescents, with a Cronbach’s alpha of 0.85 in this sample, indicating a high internal consistency.

#### 2.2.3 Training and sports-related covariates

Multiple dimensions of training load and sport participation were rigorously measured. The amount of training was quantified as the number of training hours per week from a standardized training log record. The training intensity was assessed by the subjective fatigue assessment of training sessions (21), and the calculation method was the duration of training sessions (minutes) multiplied by the training intensity (CR-10 scale score). Competition exposure includes the frequency of official competitions per month and the time (minutes) played in competitions. Sport-specific factors include field position (classified as goalkeeper, defender, midfielder, or forward), years of formal football training, and participation in other sports. A history of previous injury was defined as any injury that resulted in time loss during the 12 months prior to the start of the study period (22).

#### 2.2.4 Demographic, anthropometric and lifestyle factors

Basic demographic information includes age, sex, and geographic area. Anthropometric indicators were measured following standardized procedures: height was measured to the nearest 0.1 cm using a height meter, weight was measured to the nearest 0.1 kg using an electronic scale, and BMI was calculated as weight/height^2^ (kg/m^2^). Lifestyle factors included academic load (number of hours per week devoted to academic activities), electronic device use (average number of hours per day), caffeine intake (number of days per week), and participation in other physical activities. Psychological factors included perceived stress, measured using the 4-item version of the Perceived Stress Scale, and motivation to participate in sport, assessed by a validated scale (23).

#### 2.2.5 Quality control and data management

Several measures have been implemented to ensure data quality. Research assistants received standardized training in data collection protocols. PSQI is measured in a controlled environment to minimize interference. Injury data collection incorporates multiple verification steps, including athlete interviews, coach validation, and medical record review. Electronic data entry adopts double entry verification and logical consistency check. Data missing patterns were examined and multiple imputation was planned for variables with missing values < 5%.

### 2.3 Statistical analysis

The analysis strategy aims to achieve the research objectives through a series of statistical methods that go beyond the traditional mean model. All analyses were performed using R software (version 4.3.0), and specialized quantile regression analyses were completed using the ‘quantreg’ package.

Our primary analysis employed quantile regression to examine how the association between sleep quality and risk of injury varies across different points in the injury distribution. Unlike traditional regression, which estimates the average effect, quantile regression allows a comprehensive examination of heterogeneous associations by modeling the conditional quantiles of the outcome variable. We set up a series of models to test selected quantiles of the impairment distribution, specifically *τ* = 0.25, 0.50, 0.75, and 0.90, which were chosen to represent the low, medium, high, and very high risk spectra. Models at lower quantiles (e.g., *τ* = 0.10) were initially explored, but it was found that the high frequency of zero damage counts in our data resulted in a failure of the model to converge to a unique solution, resulting in unstable, non-interpretable estimates. Therefore, we focus our main analysis on quantiles for which robust estimation is feasible. All models were adjusted for potential confounders in a stepwise manner. The basic model takes sleep quality as the main exposure variable, and the subsequent model gradually adds demographic factors, training-related variables, lifestyle factors, and finally adds fixed effects at the team level to control the unobserved club-level heterogeneity. This approach allows us to examine heterogeneous associations and determine whether the effect of sleep quality is amplified at higher points in the injury risk distribution. To control for unmeasured confounding at the organizational level (such as coaching philosophy, training facilities, and medical support), we included club membership as a categorical variable with a dummy code to contain team fixed effects. Robust standard errors and confidence intervals were provided for all quantile-specific coefficients by bootstrap sampling with 1,000 replicates. Considering that the outcome is the number of impairments (count data), we chose linear quantile regression because its interpretation of heterogeneous associations is more intuitive and it relaxes the distributional assumptions compared to generalized linear models. We acknowledge that linear models for count data may predict unrealistic negative values; however, our primary interest is in comparing the pattern of coefficients across different quantiles (i.e., effect heterogeneity), rather than point prediction, for which linear quantile regression is a robust and widely used tool. The consistency of the findings with the alternative model settings was verified by a sensitivity analysis.

To complement the main analysis, we conducted several additional analyses to examine the robustness and nuance of the findings. We explored potential nonlinearities in the sleep-impairment association by adding restricted cubic splines to the quantile regression framework, allowing visualization of dose–response patterns without assuming linearity. Sensitivity analyses included: re-estimation of the model using alternative operationalizations of impairment outcomes (including binary outcomes (whether impairment occurred) using logistic regression analysis (Table 1), and the secondary outcome of severe injury (absence >28 days) using a binary quantile regression to determine if the heterogeneous pattern was maintained for more clinically significant events); In addition, to address concerns about the use of a linear model to handle count outcomes, we performed a sensitivity analysis using a Poisson regression model, which confirmed a positive association between PSQI and the number of injuries (incidence rate ratio: 1.12, 95% CI: 1.08–1.16, *p* < 0.001), supporting the robustness of the main finding; Different combinations of covariates were tested; interaction effects between sleep quality and key moderators, such as prior history of impairment, were examined; and the impact of potential outliers was assessed by robust regression techniques. The results of all sensitivity analyses are reported in the results section and its corresponding tables. The consistency of the results under these alternative settings will strengthen our confidence in the main conclusion that the association between sleep quality and injury risk is heterogeneous.

**Table 1:** Robustness analysis: binary logistic regression for injury occurrence.

Variable	Model 1: Basic adjustments	Model 2: Full sports model	Model 3: Comprehensive model
PSQI categories			
PSQI 0–3 (Reference)	1.00	1.00	1.00
PSQI 4–5	1.52 (0.89, 2.61)	1.48 (0.86, 2.55)	1.45 (0.84, 2.51)
PSQI 6–8	2.34^***^ (1.48, 3.70)	2.28^***^ (1.43, 3.63)	2.24^***^ (1.40, 3.58)
PSQI >8	3.52^***^ (2.24, 5.53)	3.48^***^ (2.20, 5.50)	3.45^***^ (2.18, 5.46)
Key covariates			
Previous injury	2.15^***^ (1.52, 3.04)	2.11^***^ (1.49, 2.99)	2.08^***^ (1.46, 2.95)
Weekly training hours	1.18^***^ (1.09, 1.28)	1.17^***^ (1.08, 1.27)	1.16^***^ (1.07, 1.26)
Training intensity	1.12^**^ (1.03, 1.22)	1.11^**^ (1.02, 1.21)	1.10^*^ (1.01, 1.20)
Perceived stress	–	1.15^**^ (1.04, 1.27)	1.14^**^ (1.03, 1.26)
Electronic device use	–	1.08 (0.98, 1.19)	1.07 (0.97, 1.18)
Caffeine consumption	–	1.13^*^ (1.01, 1.26)	1.12^*^ (1.00, 1.25)
Model fit statistics			
AIC	578.3	572.8	571.2
BIC	612.5	625.3	628.9
Nagelkerke *R*^2^	0.28	0.31	0.32
Hosmer–Lemeshow test	*p* = 0.423	*p* = 0.385	*p* = 0.401

Results presented as odds ratios (95% confidence intervals). All models include age, gender, BMI, and team fixed effects. ^*^p < 0.05, ^**^p < 0.01, ^***^p < 0.001.

## 3 Results

### 3.1 Sample characteristics

A total of 478 junior football players from 12 clubs were included in the final analysis. The descriptive characteristics of the total sample and the grouping by sleep quality (PSQI ≤5 vs. >5) are shown in Table 2. The sample had a mean age of 15.3 (±1.9) years and was predominantly male (68.0%). Overall, participants reported an average of 1.3 (±1.6) impairments in the previous season, with 42.9% experiencing at least one.

**Table 2:** Sample characteristics of adolescent football players (*n* = 478).

Variable	Total sample (*n* = 478)	Low PSQI group (≤5, *n* = 255)	High PSQI group (>5, *n* = 223)	*p*-value
Demographic characteristics				
Age (years)	15.3 ± 1.9	15.1 ± 1.7	15.6 ± 2.1	0.003
Male, *n* (%)	325 (68.0)	178 (69.8)	147 (65.9)	0.362
BMI (kg/m^2^)	20.4 ± 2.2	20.2 ± 2.1	20.7 ± 2.3	0.007
Sports-related characteristics				
Football experience (years)	4.9 ± 2.4	4.6 ± 2.2	5.3 ± 2.5	<0.001
Weekly training hours	8.6 ± 2.9	8.3 ± 2.7	9.0 ± 3.1	0.006
Training intensity (sRPE)	3,920 ± 1,280	3,780 ± 1,210	4,080 ± 1,320	0.008
Competition frequency (per month)	3.2 ± 1.5	3.0 ± 1.4	3.5 ± 1.6	0.001
Playing position, *n* (%)				
Goalkeeper	58 (12.1)	32 (12.5)	26 (11.7)	0.769
Defender	135 (28.2)	75 (29.4)	60 (26.9)	
Midfielder	172 (36.0)	89 (34.9)	83 (37.2)	
Forward	113 (23.6)	59 (23.1)	54 (24.2)	
Previous injury, *n* (%)	172 (36.0)	78 (30.6)	94 (42.2)	0.008
Psychological and lifestyle factors				
Perceived stress (PSS-4)	5.8 ± 2.3	5.2 ± 2.1	6.5 ± 2.4	<0.001
Academic load (hours/week)	28.5 ± 8.3	27.2 ± 7.9	30.0 ± 8.6	<0.001
Electronic device use (hours/day)	3.8 ± 1.9	3.4 ± 1.7	4.3 ± 2.0	<0.001
Caffeine consumption (days/week)	2.1 ± 1.8	1.7 ± 1.5	2.6 ± 2.0	<0.001
Sleep and injury outcomes				
PSQI global score	5.9 ± 2.7	3.8 ± 1.0	8.3 ± 1.9	<0.001
Number of injuries	1.3 ± 1.6	0.9 ± 1.2	1.8 ± 1.9	<0.001
Any injury, *n* (%)	205 (42.9)	89 (34.9)	116 (52.0)	<0.001
Severe injury, *n* (%)	72 (15.1)	28 (11.0)	44 (19.7)	0.006

Stratification by PSQI scores showed significant differences between groups. Athletes in the “high PSQI” group (poorer sleep quality, *n* = 223) were older, had a higher BMI, and had more years of football training than athletes in the “low PSQI” group (*p* < 0.01 for all comparisons). They are also under greater training and competition loads, including more training hours per week, higher training intensity and more frequent competitions. Crucially, the high PSQI group reported a significantly higher incidence of prior injury (42.2% vs. 30.6%, *p* = 0.008). The group also exhibited relatively poor psychological and lifestyle characteristics, including higher perceived stress, academic load, and daily time spent using electronic devices (*p* < 0.001 for all comparisons). As expected, injury outcomes were significantly worse in the high PSQI group, with a higher incidence of injuries (52.0% vs. 34.9%), a higher number of injuries, and a higher proportion of severe injuries.

### 3.2 Main analysis: heterogeneous association between sleep quality and injury risk

Table 3 presents the results of a quantile regression analysis examining the association of PSQI total score with injury risk across different injury distribution quantiles (*τ*). The analysis revealed a clear pattern of heterogeneity. The association between PSQI score and risk of injury was positive and statistically significant across all quantiles and showed a clear trend toward amplification — the strength of the association increased substantially at higher quantiles. The PSQI coefficient was 0.07 (95% CI: 0.01, 0.13) at the 25th quantile (*τ* = 0.25) and increased monotonically to 0.33 (95% CI: 0.23, 0.43) at the 90th quantile (*τ* = 0.90). This suggests that for high-risk adolescent athletes (at the 90th percentile of the conditional injury distribution), the expected number of injuries would increase by 0.33 for each 1-point increase in PSQI score, representing an approximately fivefold amplification of the effect strength compared to low-risk athletes (at the 25th percentile).

**Table 3:** Comprehensive quantile regression analysis of injury risk determinants.

Variable	*τ* = 0.25	*τ* = 0.50	*τ* = 0.75	*τ* = 0.90
PSQI global score	0.07^*^ (0.01, 0.13)	0.14^***^ (0.07, 0.21)	0.23^***^ (0.15, 0.31)	0.33^***^(0.23, 0.43)
Demographic factors				
Age	0.03 (−0.02, 0.08)	0.05^*^ (0.01, 0.09)	0.08^**^ (0.03, 0.13)	0.11^***^(0.05, 0.17)
Gender (Male)	−0.04 (−0.11, 0.03)	−0.07^*^ (−0.14, 0.00)	−0.11^**^ (−0.20, −0.02)	−0.16^***^(−0.27, −0.05)
BMI	0.02 (−0.03, 0.07)	0.04 (0.00, 0.08)	0.06^*^ (0.01, 0.11)	0.08^**^ (0.02, 0.14)
Sports-related factors				
Football experience	0.04 (−0.01, 0.09)	0.07^**^ (0.02, 0.12)	0.10^***^ (0.04, 0.16)	0.15^***^ (0.08, 0.22)
Weekly training hours	0.05^*^ (0.00, 0.10)	0.08^***^ (0.03, 0.13)	0.13^***^ (0.07, 0.19)	0.19^***^ (0.12, 0.26)
Training intensity (sRPE/100)	0.03 (−0.02, 0.08)	0.05^*^ (0.01, 0.09)	0.07^**^ (0.02, 0.12)	0.11^***^ (0.05, 0.17)
Competition frequency	0.04 (−0.01, 0.09)	0.06^**^ (0.01, 0.11)	0.09^***^ (0.03, 0.15)	0.13^***^ (0.06, 0.20)
Playing position (ref: goalkeeper)				
Defender	0.04 (−0.05, 0.13)	0.06 (−0.03, 0.15)	0.08 (−0.02, 0.18)	0.10 (−0.02, 0.22)
Midfielder	0.06 (−0.03, 0.15)	0.08 (−0.01, 0.17)	0.10 (0.00, 0.20)	0.12 (0.00, 0.24)
Forward	0.05 (−0.04, 0.14)	0.07 (−0.02, 0.16)	0.09 (−0.01, 0.19)	0.11 (−0.01, 0.23)
Previous injury	0.11^**^ (0.03, 0.19)	0.17^***^ (0.09, 0.25)	0.26^***^ (0.16, 0.36)	0.38^***^ (0.25, 0.51)
Psychological and lifestyle factors				
Perceived stress	0.04 (−0.01, 0.09)	0.07^**^ (0.02, 0.12)	0.11^***^ (0.05, 0.17)	0.15^***^ (0.08, 0.22)
Academic load	0.02 (−0.03, 0.07)	0.04 (0.00, 0.08)	0.06^*^ (0.01, 0.11)	0.09^**^ (0.03, 0.15)
Electronic device use	0.03 (−0.02, 0.08)	0.05^*^ (0.01, 0.09)	0.08^**^ (0.03, 0.13)	0.11^***^ (0.05, 0.17)
Caffeine consumption	0.05^*^ (0.00, 0.10)	0.07^**^ (0.02, 0.12)	0.10^***^ (0.04, 0.16)	0.14^***^ (0.07, 0.21)
Team fixed effects	Controlled	Controlled	Controlled	Controlled
Pseudo *R*^2^	0.21	0.27	0.34	0.41

Coefficients represent the expected change in injury count. 95% confidence intervals in parentheses. ^*^p < 0.05, ^**^p < 0.01, ^***^p < 0.001.

Several covariates also showed heterogeneous effects. The history of previous injury showed the largest gradient, with the coefficient increasing from 0.11 to 0.38. Training-related variables (hours per week, intensity, frequency of competition) and lifestyle factors (perceived stress, caffeine intake) also showed a progressively stronger association with injury risk at higher quantiles. Pseudo-R ^2^ values increased from 0.21 to 0.41, indicating a better fit of the model to explain injury risk in high-risk athletes.

### 3.3 Visualization of heterogeneous effects and dose–response relationship

**Figure 1:**
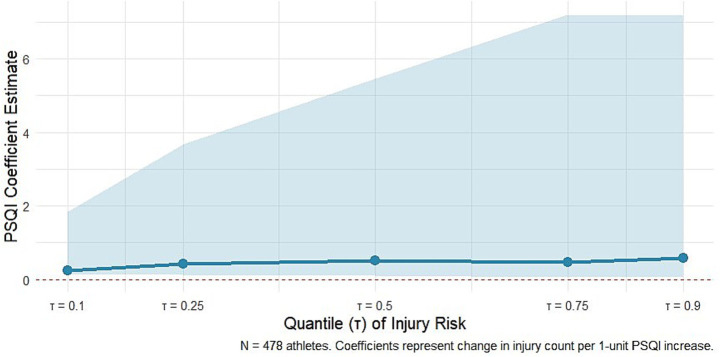
Trend of PSQI coefficient with quantile of injury risk. This figure shows the trend of PSQI coefficient estimates at different quantiles of the injury risk distribution. Positive coefficients indicate that poorer sleep quality (higher PSQI scores) is associated with increased risk of impairment. The upward trend suggests that the effect of sleep quality is stronger for athletes at higher baseline injury risk (right side). Error bands indicate 95% confidence intervals based on bootstrap estimates.

The core findings of the heterogeneity effect are presented graphically in Figure 1, which plots the changes in the estimates of the PS QI coefficients with respect to the quantiles of the impairment distribution (*τ* = 0.05 to 0.95). The graph confirms a clear, monotonically increasing trend, indicating a deleterious association between poor sleep quality and injury risk, which is progressively stronger for adolescent athletes with higher baseline risk. Confidence interval bands narrow at higher quantiles, indicating more precise estimates of this enhancement effect in the most vulnerable athletes.

**Figure 2:**
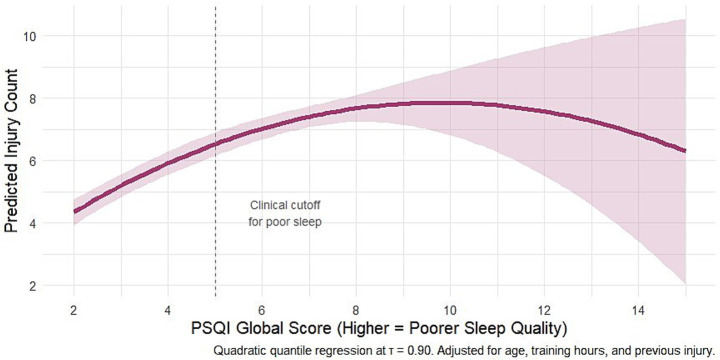
Dose–response relationship between total PSQI score and predicted number of injuries in high-risk athletes. This graph depicts the dose–response relationship between the total PSQI score and the predicted number of injuries at the 90th quantile (high-risk athletes). The curve shows a non-linear relationship: the risk of impairment rises more rapidly at higher PSQI scores, especially after exceeding the clinical cut-off for poor sleep quality (PSQI >5, dotted line). This prediction is based on a quadratic quantile regression model adjusted for covariates.

Figure 2 depicts the non-linear dose–response relationship between the continuous PSQI total score and the number of predicted impairments at the 90th quantile (*τ* = 0.90). The curve revealed a J-shaped association, with the risk of impairment being relatively stable at lower PSQI scores (good sleep quality), but beginning to accelerate significantly beyond a score of about 6. This threshold effect intuitively emphasizes that the transition from moderate to poor sleep quality has disproportionately high consequences for the injury risk of high-risk athletes.

**Figure 3:**
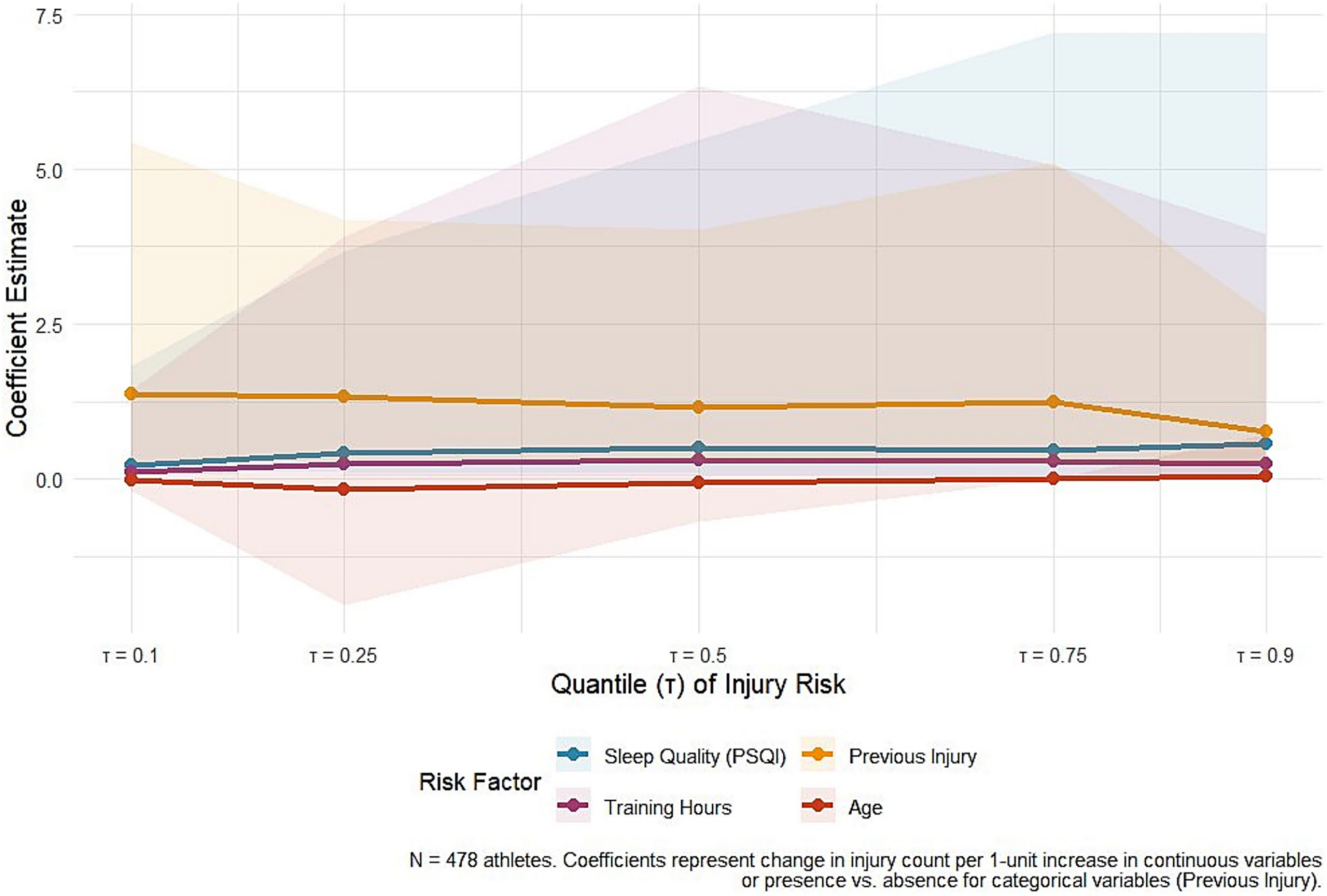
Comparison of quantile regression coefficients for key risk factors. This figure compares the quantile regression coefficients of four key risk factors on the injury risk distribution. All variables showed heterogeneous effects, with sleep quality and history of previous impairment showing the most significant gradient changes. The steeper slopes of these variables at higher quantiles suggest that their importance is more pronounced for athletes who are already at higher risk of injury.

Figure 3 presents a comparative analysis of the quantile specificity coefficients for the four key risk factors. Although all variables showed some degree of heterogeneity, the gradient change in PSQI scores was among the steepest, second only to prior injury history. This comparative visualization reinforces the idea that sleep quality is not just a risk factor, but a particularly potent one for athletes who are predisposed to injury due to other intrinsic and extrinsic factors.

### 3.4 Robustness checks and sensitivity analyses

Table 1 shows the results of the robustness analysis performed using the alternative model settings. Binary logistic regression models confirmed the main findings, showing a consistent dose–response relationship between PSQI scores and the probability of impairment. The odds ratio of injury in athletes with the highest PSQI classification (>8) was 3.45 times higher than that in the control group (95% CI: 2.18–5.46).

To further assess the robustness of our findings to more clinically meaningful outcomes, we repeated the analysis with an endpoint of severe injury (absence >28 days). In addition to confirming the overall association, we tested whether a pattern of effect heterogeneity characterized by stronger effects in high-risk situations was maintained. The PSQI score coefficient was not significant at lower quantiles (e.g., *τ* = 0.25, *β* = 0.01, *p* = 0.232) but became statistically significant and greater at the 90th quantile (*β* = 0.08, 95% CI: 0.02, 0.15; *p* = 0.009), reflecting the pattern of amplification of any temporal loss lesion.

Sensitivity analyses using different combinations of covariates and model settings yielded consistent results. The PS QI-impairment association remained robust across all sensitivity tests, with minimal change in effect size or significance. Models that excluded psychological and lifestyle covariates produced nearly identical PSQI coefficients, suggesting that these factors act through different pathways than sleep quality.

## 4 Discussion

In this study, we used quantile regression to go beyond the traditional mean-based analysis approach, thereby providing a nuanced understanding of how sleep quality is associated with injury risk across the adolescent football player population. Our core findings reveal a significant heterogeneity: the adverse association between poor sleep quality and risk of injury, while statistically significant for all, is not fixed, but shows a quantile-dependent gradient, showing a clear amplification effect. This effect gradually increased with the distribution of injuries and was strongest in high-risk athletes at the 90th percentile. This model fundamentally challenges the traditional “one-size-fits-all” concept of sleep as an evenly acting risk factor and provides quantitative evidence for its role as a potent amplifier of existing vulnerabilities. The main contribution of this work is not to confirm that sleep is a risk factor, but to reveal and quantify its differential impact, indicating that its effect size depends on the existing risk characteristics of athletes.

### 4.1 Mechanistic interpretation of the amplified association

#### 4.1.1 Physiological pathways of vulnerability amplification

The disproportionate impact of poor sleep quality on high-risk athletes can be understood through interrelated physiological mechanisms, which we call “vulnerability amplification.” While previous studies have shown that sleep deprivation impairs muscle recovery and increases systemic inflammation in the general athlete population (24, 25), our results extend this knowledge by demonstrating that these associations are not uniform. High-risk athletes often train at physiological limits, characterized by elevated steady-state loads due to cumulative training stress, subclinical inflammation, or the sequelae of previous injury. This concept is supported by recent studies showing that athletes with high chronic training loads exhibit endocrine and inflammatory dysregulation, making them more vulnerable to the negative effects of various stressors (26). When impaired sleep disrupts the hormonal milieu, reduces growth hormone secretion, elevates cortisol levels, and alters the inflammatory cytokine profile, the capacity for cellular repair and neuromuscular recovery becomes severely deficient (27). This creates a “perfect storm” in which athletes who already have physical vulnerabilities experience exponentially worse consequences from sleep disorders than their healthier peers.

#### 4.1.2 Neurocognitive mechanisms and threshold effects

Heterogeneous associations were observed in the injury risk distribution, further reflecting key neurocognitive pathways. Our findings complement and extend the emerging evidence on sleep and injury risk. A recent prospective study of adolescent athletes further emphasized that sleep is an independent risk factor for injury, reinforcing the underlying relationship we sought to explore more deeply (28). Sleep deprivation consistently impairs prefrontal cortex function, resulting in deficits in executive function, including attentional regulation, error monitoring, and risk assessment (29). For high-risk athletes who may already exhibit mild neuromuscular control deficits or risk-taking tendencies, the additional cognitive impairment from poor sleep may be sufficient to cross a threshold where proper motor technique and damage avoidance behavior are compromised (30). This neurocognitive explanation is particularly important in football, where momentary decisions in the course of a change of direction or landing sequence determine the outcome of an injury. The cumulative effects of slower reaction times, reduced situational awareness, and impaired motor learning (all consequences of poor sleep (31)) are most strongly associated with injury risk in athletes who are already vulnerable. It is likely that neurocognitive pathways account for a portion of the strong associations we observed at the higher quantiles of injury risk.

#### 4.1.3 Behavioral pathways and bidirectional relationships

Behavioral mechanisms may further exacerbate these physiological and cognitive associations, creating a self-reinforcing risk cycle (32). Our findings build on a growing body of literature that explores the multifaceted benefits of sleep for athletes. Contemporary studies not only confirm that sleep optimization can improve exercise performance, but also emphasize its role in improving physiological and cognitive indicators that are crucial for recovery and injury resistance (7, 33). Specifically, adequate sleep is associated with enhanced metabolic recovery, a more positive psychological state, and improved cognitive function, which underlie strong injury resistance (34). Athletes with poor sleep often show reduced motivation to perform appropriate warm-up activities, reduced adherence to strength and conditioning programs, and altered perception of pain. These behavioral changes may be more strongly associated with injury in athletes who are already at higher risk (35, 36). Moreover, the relationship between sleep and injury is likely to operate in a complex network of bidirectional influences, in which pain from previous injury disrupts sleep quality (37), possibly creating a vicious cycle that is particularly difficult for high-risk individuals to escape (38). The possibility of such a two-way relationship underscores why our cross-sectional findings must be interpreted as an association, rather than a causal relationship, which helps explain the persistence of injury risk in certain subgroups of athletes and highlights the need for a comprehensive approach to intervention (17).

#### 4.1.4 Musculoskeletal considerations specific to young athletes

The amplification observed in our 12- to 18-year-old sample is also indicative of physiological factors specific to adolescence. This age coincides with the period of rapid growth in adolescents, a period of rapid skeletal growth that often exceeds the adaptive capacity of the tendon unit, resulting in temporary deficits in flexibility and neuromuscular control. As mentioned above, neurocognitive impairment caused by poor sleep may be superimposed on this already fragile “maturity mismatch.” The additional deterioration of reaction time, motor coordination, and executive function caused by sleep deprivation will disproportionately impair the ability of adolescents to maintain dynamic joint stability during high-speed soccer, thereby significantly increasing the risk of injury (39). This mechanism provides a strong, biologically plausible explanation for why the “vulnerability amplification” effect is particularly pronounced at this age.

### 4.2 Methodological and theoretical implications

Our findings have important methodological and theoretical implications for exercise epidemiology. The significant heterogeneity we observed suggests that traditional mean-based methods, while valuable in identifying universal associations, may systematically underestimate the true strength of the association between sleep quality and impairment in vulnerable subpopulations. By failing to account for effect heterogeneity, mean-based models can obscure the most critical public health insight: identifying vulnerable subpopulations where interventions are likely to have the greatest absolute benefit (40). The quantile regression approach we employed provides a powerful alternative for detecting this heterogeneity and identifying the subpopulations that are most likely to benefit from targeted interventions (13), thus providing a methodological path towards the conceptual framework of “precision injury prevention” (41).

Theoretically, our results support and extend the “cumulative risk” model of injury etiology advocated by Bittencourt et al. in their complex systems approach to sports injuries (42). Sleep quality does not seem to act in isolation, but interacts with existing vulnerabilities to determine the outcome of injury. Our finding of a significant gradient provides quantitative evidence for this complex interaction, suggesting that poor sleep not only increases risk, but also exponentially for those individuals who are already at risk. This view is consistent with the complex systems approach to injury prevention, which emphasizes the interrelatedness of risk factors and the importance of identifying key leverage points in the risk profile of individual athletes (43).

### 4.3 From universal prevention to precision practice: the value of effect heterogeneity

The most prominent significance of our research results lies in the transformation from knowledge to practice. While the universal importance of sleep is well established, our study provides the empirical evidence necessary to drive the shift from universal, “one-size-fits-all” recommendations to efficient, targeted practices (44). The observed five-fold amplification of the sleep effect in high-risk athletes is not just a statistical nuance, but a clear signal of resource allocation. This suggests that sleep intervention is not equally effective for all individuals and should be considered as a highly leveraged tool that yields the greatest return on investment when directed to the most vulnerable subpopulations (45).

At a practical level, our findings suggest that optimizing injury prevention programs can begin by identifying high-risk athletes (for example, by screening for past injury history, high training loads, or other risk factors) and then prioritizing sleep assessment and intervention for that group. This tiered approach maximizes the preventive benefits of sleep health promotion, making public health actions more effective and sustainable (46).

### 4.4 Research limitations and methodological considerations

There are several limitations to consider when interpreting our findings. First and foremost, the cross-sectional retrospective design (sleep quality was assessed after the past season’s impairment outcomes) poses a fundamental challenge to causal inference. The possibility of reverse causation (i.e., adverse effects of prior impairment on subsequent sleep quality) cannot be ruled out. Although the PSQI assessment of sleep in the past month may predate some recent impairments, the biological plausibility of our findings, while supportive, does not fully overcome this design limitation. Future prospective studies are needed to confirm the chronological order. Second, self-reported sleep and impairment data, while convenient for large-scale studies, may be subject to measurement error and recall bias. While our validation process mitigates this, the accuracy of injury recalls, particularly minor injuries throughout the season, remains a limitation. In addition, our assessment of training load was limited to subjective sRPE methods, lacking objective external load data (e.g., GPS-derived running distance, high-intensity acceleration/deceleration). This may introduce unmeasured residual confusion about the true mechanical stress experienced by the athlete.

Although we controlled for a comprehensive set of covariates and used fixed effects to deal with team-level heterogeneity, as with any observational study, the potential for residual confounding remains. Genetic susceptibility, intrinsic motivation, or unmeasured environmental factors, among others, may partially explain the observed association. In addition, our sample, while substantial, may provide less precise estimates at the two end quantiles of the distribution (especially for subgroup analyses) because the estimate of the 90th quantile relies on a smaller subset of data. Moreover, the high frequency of zero-injury outcomes prevented the convergence of quantile regression models at very low quantiles (e.g., *τ* = 0.10). Finally, the use of linear quantile regression for count results, while justified by its strength in explaining heterogeneous associations, may be seen as a limitation. However, we confirmed the robustness of the core finding of heterogeneous association by using logistic regression to analyze binary injury results, severe injury results, and Poisson regression to analyze sensitivity analysis of count results. In addition, testing for multiple quantiles and covariates increases the risk of Type I error due to multiple comparisons. Therefore, the focus of our interpretation remains on the consistent pattern and magnitude of the coefficient gradients, rather than significance of any individual coefficient. We believe that the monotonically increasing, dose–response-like pattern of change across quantiles of the PSQI coefficient is unlikely to be entirely due to chance.

## 5 Conclusion

This study shows that while poor sleep quality is universally associated with increased injury risk in adolescent football players, this association is significantly heterogeneous, showing an amplification effect that is concentrated in a vulnerable subgroup of high-risk athletes. The adverse association of poor sleep quality gradually increased with the distribution of injury risk, peaking in athletes at the 90th percentile. These findings challenge the traditional understanding of sleep as a risk factor for uniform action and provide a quantitative evidence base for precision-based approaches in sports injury prevention. However, given the methodological limitations of the cross-sectional design, these results should be interpreted as revealing strong and heterogeneous associations rather than causal effects. Future studies should give priority to longitudinal and interventional designs to confirm the temporal sequence and potential causal nature of this relationship, and to evaluate the effectiveness of targeted sleep interventions for high-risk adolescent athletes.

## Data Availability

The raw data supporting the conclusions of this article will be made available by the authors, without undue reservation.
